# Physiotherapeutic Approach for Paraparesis Resulting From L2-S1 Disc Bulge Along With Diabetic Neuropathy: A Case Report

**DOI:** 10.7759/cureus.52056

**Published:** 2024-01-10

**Authors:** Reva Rajurkar, Pallavi Harjpal, Priya Tikhile

**Affiliations:** 1 Neuro-Physiotherapy, Ravi Nair Physiotherapy College, Datta Meghe Institute of Higher Education and Research, Wardha, IND

**Keywords:** sensory reeducation, rehabilitation, paraparesis, diabetic neuropathy, disc bulge

## Abstract

A degenerative disc disease is characterized by osteophytes, disc space reduction, nerve compression and discomfort are all symptoms of disc bulging. Diabetic neuropathy, a condition leading to significant health issues, involves a sensory dysfunction initiating in the lower extremities and progressing to pain. In the present case, a 61-year-old retired physical training teacher came to the hospital with complaints of difficulty in walking, sitting, and standing in the past two years. The patient also complained of a tingling sensation in the upper and lower limbs, low back pain, and body stiffness. The patient has a known case of intervertebral disc prolapse at L2-S1 level, two years back. Patient had a history of diabetes mellitus type 2, hypertension, and hypothyroidism for the past three years. The study delves into the detailed evaluation, customized treatment plan, and rehabilitation strategies employed to meet the patient's condition. It emphasizes the value of a multidisciplinary approach, including physical therapy, in the treatment of complicated musculoskeletal and neurological illnesses, intending to improve the patient's mobility, functionality, and overall quality of life.

## Introduction

Lumbar disc degeneration refers to the deterioration of the discs safeguarding the vertebrae. It can manifest at various levels, with the L3-L4 and L4-S1 vertebrae being the most frequently affected [1,2]. The condition causes osteophytes, disc space loss, disc protrusion, nerve pain, and degenerative disc degeneration [3]. Degenerative stenosis, also known as canal constriction, is characterised by gradual degenerative changes in the disc, facet joints, and surrounding soft tissue [4]. Because each lumbar disc contacts with up to three pairs of spinal roots, disc degeneration can cause irritation of the adjacent nerve roots [5]. While usually associated with neuropathic pain and neurological signs, significant nerve injury may result in a pain syndrome [6]. Age, financial situation, vertebral stress, obesity, tobacco use, rigorous lifting, vibration, trauma, immobility, psychological elements, gender, height, genetics, and hereditary traits are all risk factors for developing lumbar disc degeneration disease and associated lumbosacral nerve compression. Certain vocations, such as industrial driving, carpentry, and office work, are also associated with increased risk factors [4,7].

In a clinical setting, degenerative lumbar disc lesions affect the lumbosacral plexus, affecting the plexus of the sacrum differently than the lumbar plexus. Lumbar plexus lesions can cause problems involving the iliohypogastric, genitofemoral, ilioinguinal, femoral, and obturator nerves [8]. Injuries to the lumbar plexus can cause impaired hip flexion, knee extension, thigh adduction, and diminished sensation in the lower abdomen, inguinal region, in addition to the entire medial, lateral, and anterior surfaces of the thigh and medial lower leg. Usually, lumbar plexus injuries cause a reduced or non-existent knee jerk reflex [9]. Sacral plexus lesions, like lumbar plexus lesions, cause pain, decreased cutaneous sensation, and muscle weakness in areas in which the gluteal, sciatic, tibial, peroneal, and pudendal nerves, as well as sacral plexus branches, are distributed. Lower extremity muscle weakness is a common complication, affecting both leg and foot muscles supplied by the peroneal and tibial nerves, as well as hip extension (gluteus maximus), hip abductors, and internal rotators (gluteus medius and tensor fasciae latae). The whole leg, the anterolateral and dorsal portions of the leg beneath the knee, and the back of the thigh may be affected by this weakness. In addition, the ankle jerk reflex might be reduced or missing [4].

Diabetic nephropathy (DN) stands out as a prevalent and impactful complication of diabetes mellitus, elevating the risks of morbidity and mortality among individuals with diabetes [10]. Diabetic nephropathy is a microvascular consequence of type 2 diabetes mellitus that destroys the small blood capillaries of the kidneys, limiting renal function. It is distinguished by a decrease in protein in the urine. Renal impairment progresses in these people from a slight functional drop to mild, moderate, or severe nephron loss [11]. Diabetic neuropathy leads to severe illness and a decline in sensory function that initiates in the lower limbs and is accompanied by discomfort [12]. DN occurs after a long period of inactivity in around one-third of diabetics. It is controversial whether patients should be tested for DN or monitored for microalbuminuria as part of the personalized medicine strategy in order to target resources for more intense treatment and early preventive treatments exclusively to those who are most at risk [13,14]. Nonproteinuric and DN without retinopathy are more common in patients with type 2 diabetes. Deciding when to begin intensive therapy might be difficult in the absence of albuminuria because renin-angiotensin system (RAS) blockade therapy is often initiated only in cases of chronic albuminuria [13]. Diabetes and nephropathy have emerged as new challenges for healthcare providers and individuals who provide care for the affected. Age, male sex, long-term diabetes, early hyperfiltration indicated by an estimated glomerular filtration rate (GFR) (90 mL/min/1.73 m2), a systolic blood pressure greater than 130 mmHg, and chronic proteinuria with concomitant retinopathy are risk factors for DN and its progression. People's quality of life, ability to work physically, and frailty are all negatively impacted when they experience weariness, discomfort, dyspnea, sarcopenia, and renal anaemia [11].

## Case presentation

Patient information

A 61-year-old male patient, who retired from his job in 2020 as a physical training teacher in school, presented to the hospital on 21/09/23 with complaints of low back pain while performing bending activities, which gradually progressed into difficulty in walking, sitting, and standing in the past two years. The patient also complained of a tingling sensation in lower limbs since six months. The patient is a known case of intervertebral disc prolapse at L2-S1 level, two years back for which he visited local practitioner where he treated conservatively. He has a history of diabetes mellitus type 2 for which he was on insulin treatment, hypertension for which he was given antihypertensive drugs, and hypothyroidism for which he was taking levo-T for the past three years. The patient lived in a rural place and was literate. Patient was dependent on caregiver for the daily living activities.

Clinical findings

The patient was well-oriented, cooperative, and conscious. Everything in the vitals was fine. There was no postural issue discovered during a physical examination. Both the lower limbs and the upper limb had impaired superficial sensations (Table 1). On motor evaluation, neither the upper nor lower limbs had any tone abnormalities. The range of motion of the lower limb was reduced (Table 2), while both upper limbs had a normal full range of activities. The deep tendon reflexes, i.e., the knee jerk and ankle jerk, were diminished in both lower extremities (Table 3). Patient was unable to stand without support.

**Table 1:** Sensory examination

Superficial Sensation	Right	Left
Light touch	Impaired	Impaired
Pin Prick	Impaired	Impaired

**Table 2:** Range of motion of both lower limbs

Joint Motion (in degree)	Right	Left
Hip flexion	0-50	0-30
Hip extension	0-15	0-10
Hip abduction	0-35	0-35
Hip internal rotation	0-25	0-10
Hip external rotation	0-25	0-15
Knee flexion	0-70	0-50
Knee extension	70-0	50-0

**Table 3:** Deep tendon reflexes Clinical Day 1 "++"  normal reflex "+" Diminished Reflex

Reflexes	Biceps jerk	Triceps jerk	Supinator jerk	Knee jerk	Ankle jerk
Right	++	++	++	+	+
Left	++	++	++	+	+

Clinical diagnosis

**Figure 1:**
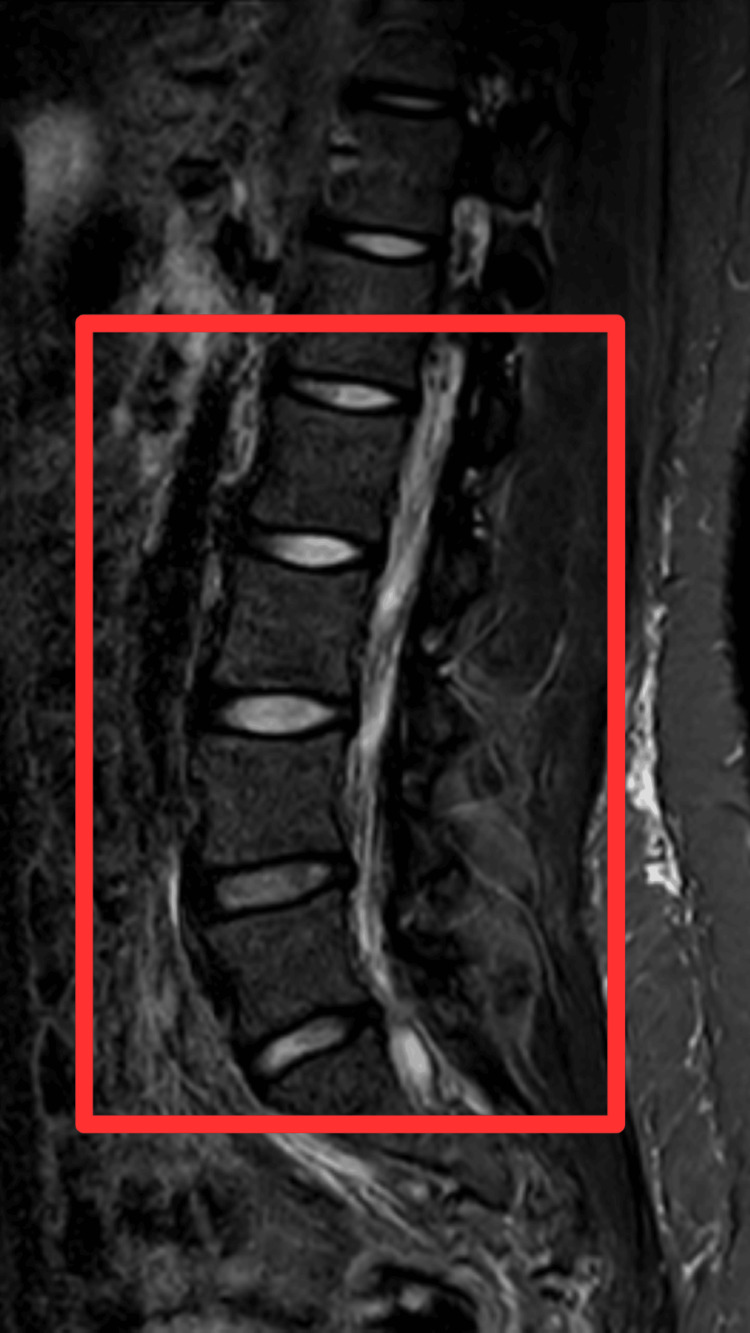
MRI findings of patient MRI findings show mild asymmetrical disc bulge at L1-2 level and mild disc bulge at L2-3 level, disc desiccation, and diffuse circumferential disc bulge causing mild bilateral neural foraminal narrowing at L3-4 level, it indents anterior thecal sac and abuts bilateral traversing L4 nerve roots, mild decreased disc height, and diffuse circumferential disc.

The patient was examined for a standard examination, an MRI, and a complete blood count (CBC). Spondylitis changes were revealed by an MRI study of the lumbosacral spine (Figure 1), which revealed mild asymmetrical disc bulge at L1-2 level and mild disc bulge at L2-3 level, disc desiccation, and diffuse circumferential disc bulge causing mild bilateral neural foraminal narrowing at L3-4 level, it indents anterior thecal sac and abuts bilateral traversing L4 nerve roots, mild decreased disc height, and diffuse circumferential disc. CBC study showed the blood sugar pre-meal at 140 mg/dL and post-meal at 220 mg/dL. After all the investigations and the clinical findings, the patient was diagnosed with paraparesis with diabetic neuropathy. Age-related atrophic changes with muscle weakness were found.

Physiotherapy interventions

**Figure 2:**
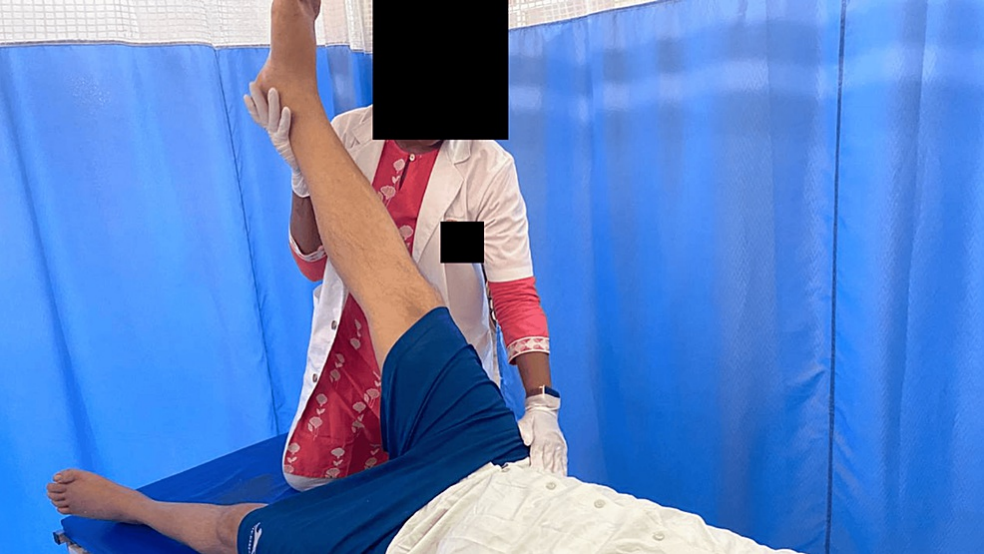
Hamstring stretching

**Figure 3:**
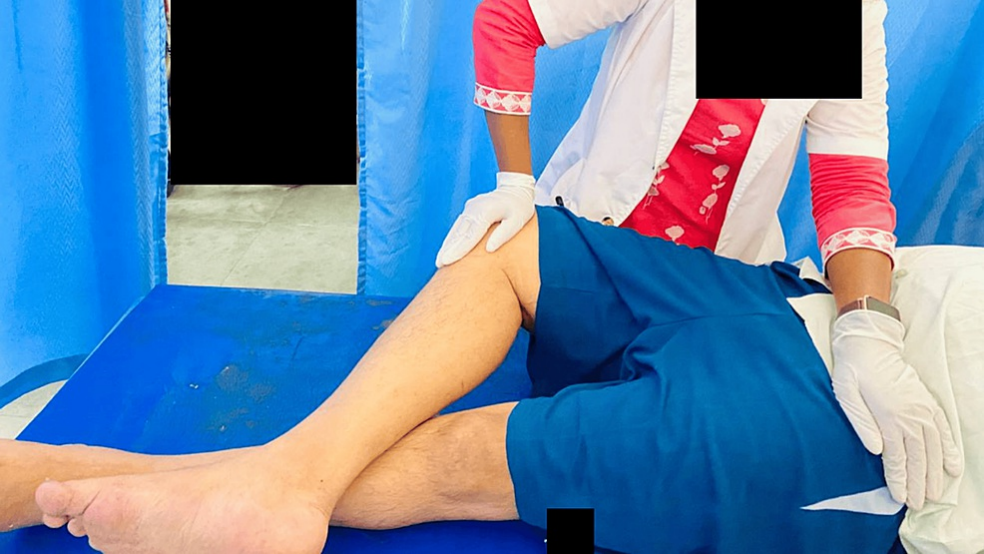
Piriformis stretching

**Figure 4:**
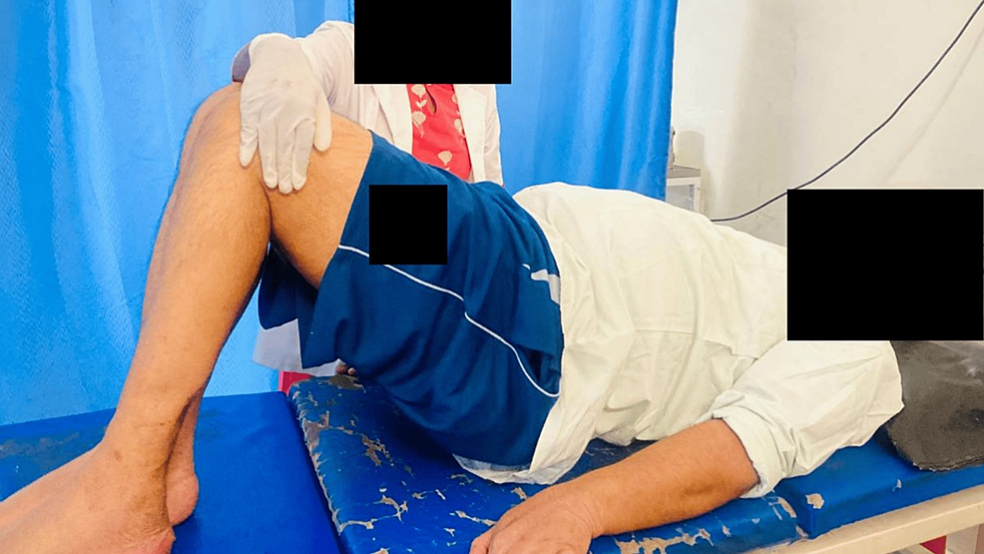
Pelvic bridging

**Figure 5:**
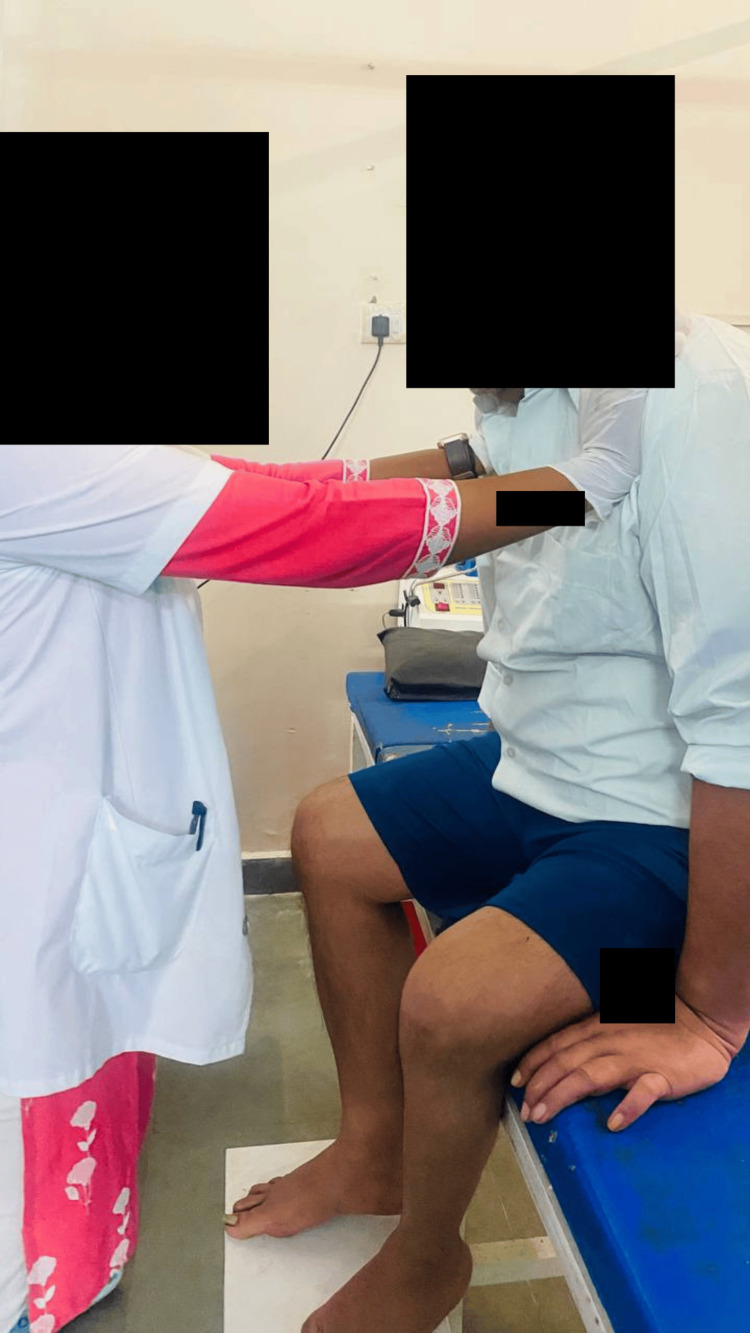
Trunk PNF (rhythmic stabilization) PNF: Proprioceptive Neuromuscular Facilitation

**Figure 6:**
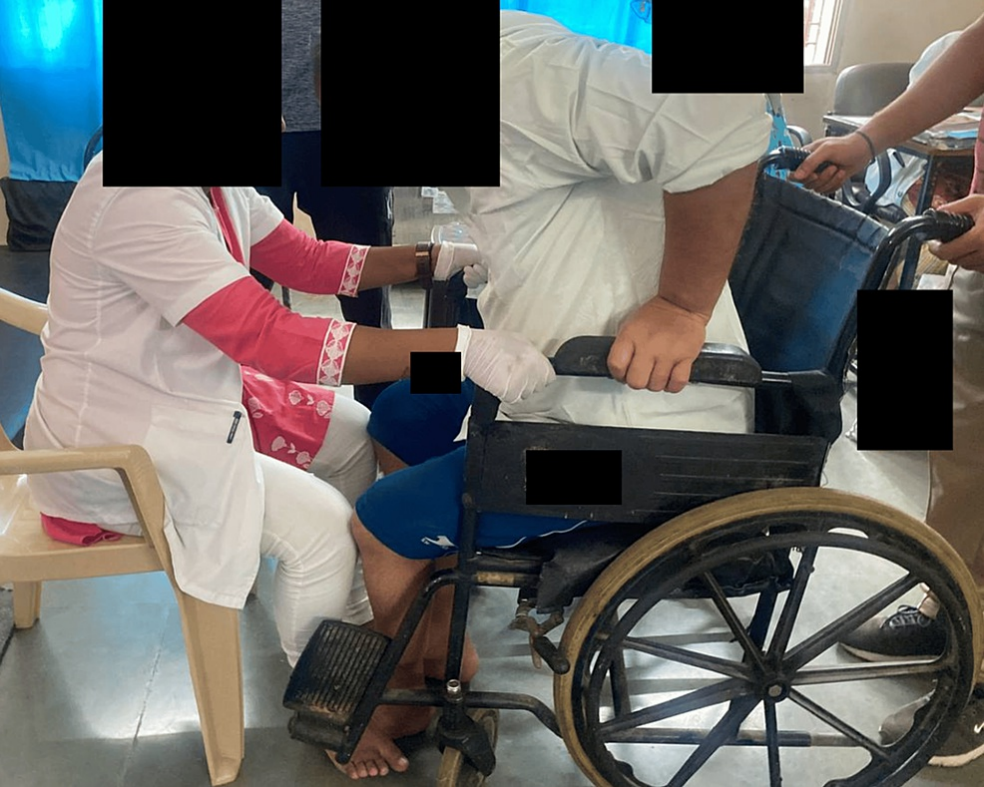
Sit to stand with minimum support (patient initiating standing)

**Figure 7:**
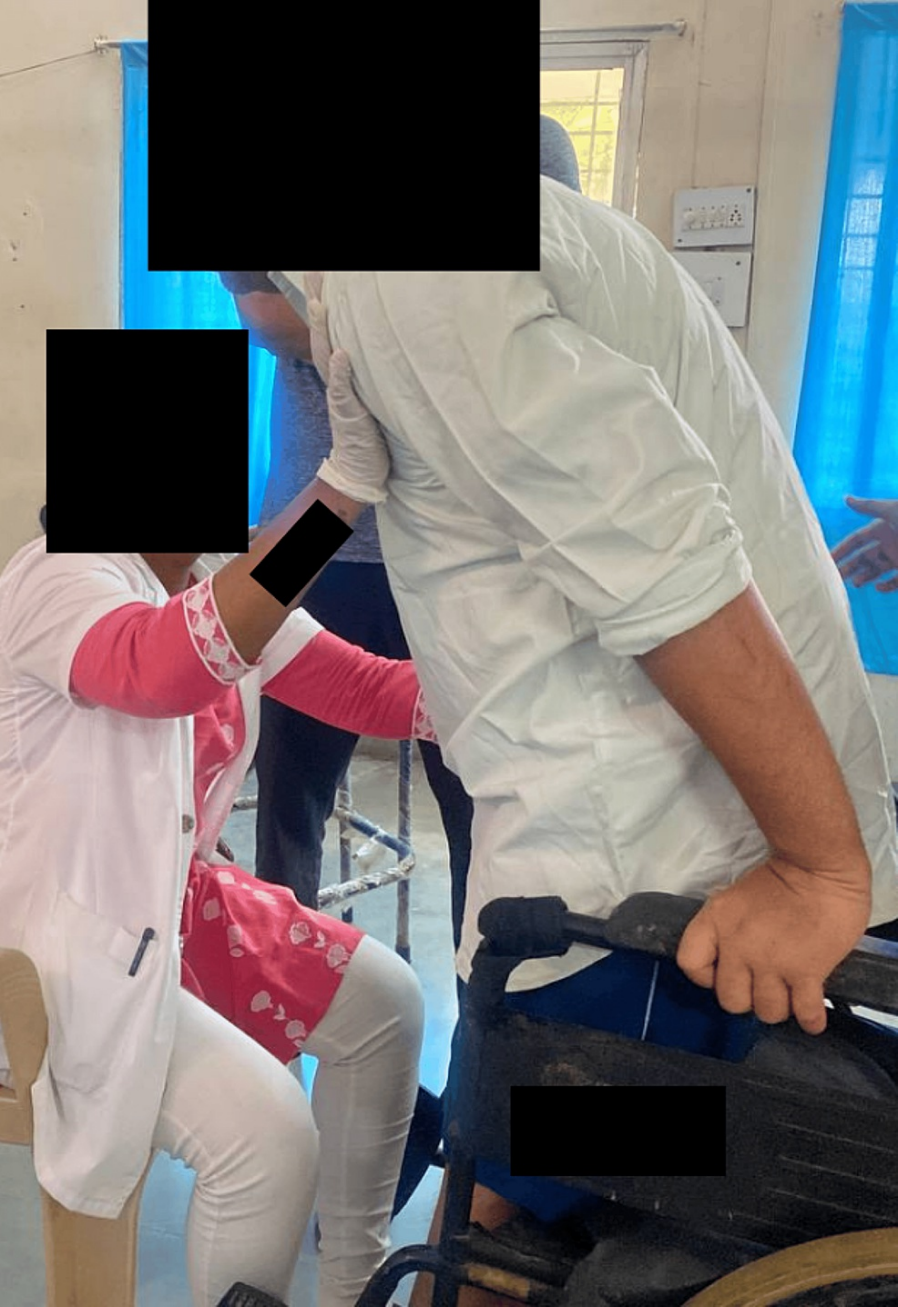
Patient able to stand with support

Physiotherapy was primarily focused on balance training, gait training, and sensory re-education. Physiotherapy care was provided once a day for one hour, which included active-assisted and passive stretching exercises for lower limbs (Figures 2, 3), pelvic bridging (Figure 4), spinal rotations, trunk proprioceptive neuromuscular facilitation (PNF; stabilizing reversals and rhythmic stabilization) (Figure 5), weight shift, reach out, sit to stand (Figures 6, 7), spot marching and gait training in parallel bars. For upper extremities, strength training was started. For sensory reeducation, brief ice massage and texture discrimination was done. Every task was executed at a pace to avoid fatigue and in alignment with the patient's comfort. Table 4 contains the treatment details. 

**Table 4:** Treatment details ROM: Range of Motion, AAROM: Active-assisted range of motion, sec: Second, reps: repetitions, PNF: Proprioceptive Neuromuscular Facilitation

Problem	Cause	Therapeutic goals	Treatment
Reduced ROM in lower limb.	Weakness of lower limb muscles due to intervertebral disc prolapse.	Maintain joint integrity and available ROM	AAROM exercise. Selective stretching to improve function (for stretching, the protocol was five reps of 2 sets with 15 sec hold with two minutes of rest between the sets).
Low back pain	Lumbar disc prolapses and immobility.	Reduce pain and improve paraspinal strength.	Static back 10 repetitions with 5 sec hold. Pelvic bridging 10 repetitions. Spinal rotation 10 repetitions on both sides.
Difficulty in maintaining balance in sitting and standing.	Lower limb and paraspinal muscle weakness and immobility.	Improve static and dynamic balance, maintain trunk stability.	Weight shift side to side. Trunk PNF, stabilizing reversals and rhythmic stabilization (in sitting), reach out in all directions. Perturbation in all directions. Sit to stand (it started with support and progressed to without support).
Difficulty in walking	Lower limb and paraspinal muscle weakness and immobility.	Maintain standing balance, gait training.	Standing within parallel bars. Initiate with spot marching and progress to walking between the bars. Progression to tandem walking, side walking, walking between obstructions, and step training.
Reduced strength	General weakness	Maintain available muscle strength	Strengthening exercises for upper extremities (it started with 1 kg of weight, progressing to 2 kg by the next 15 days).
Sensory impairment in the lower limb	Diabetic neuropathy	Sensory reeducation	Brief ice massage. Texture discrimination.
Patient and caregiver education	-	-	Explain the home exercise program in easy language. Explain all warning signs, do’s and don’ts.

Follow-up and outcome measures

After 21 days of physical therapy intervention, there was a notable enhancement in both the sensory and motor statuses of the patients, coupled with an increased range of motion (ROM) in both lower limbs (Table 5). Deep tendon reflexes were found in both lower limbs, including the knee jerk and ankle jerk. Additionally, all superficial sensations remained intact in the bilateral upper and lower limbs. The patient demonstrated improved balance by being able to sit at the bedside and stand without support. Furthermore, the patient could initiate walking with assistance. Outcome measures for day 1 and follow-up day 21 are given in Table 6.

**Table 5:** Range of motion of both the lower limbs on follow-up day

Joint motion (in degree)	Right	Left
Hip flexion	0-100	0-100
Hip extension	0-25	0-20
Hip abduction	0-40	0-40
Hip internal rotation	0-40	0-40
Hip external rotation	0-40	0-40
Knee flexion	0-110	0-110
Knee extension	110-0	110-0

**Table 6:** Outcome Measures

Outcome Measures	Day 1	Day 21
Barthel index	55/100	75/100
Berg Balance Scale	5/56	21/56
Functional Independence Measure	131/210	180/210

## Discussion

Degeneration of the lumbar discs in the backbone causes a variety of symptoms, including pains, neuropathic issues, or even nerve damage. This condition is known as lumbar disc degeneration. There are a variety of risk factors associated with this situation, including age, socioeconomic standing, smoking, obesity, and workplace hazards [3]. The clinical presentation of lumbar disc degeneration, in particular its potential effects on the lumbar and sacral plexuses, can result in a variety of symptoms, such as weakness, loss of sensation, and changes in reflexes [15]. It is made evident how participation of the sacral plexus differs from the lumbar region [4]. Diabetic neuropathy is a major complication of diabetes mellitus. Kidney damage is a defining feature, resulting in the loss of urine protein and the progressive deterioration of renal features. The text poses the topic of whether screening for early nephropathy symptoms should be done in order to allow for prompt intervention [16]. The importance of the renin-angiotensin system (RAS) blockade treatment is emphasized because it is specifically started after the development of chronic albuminuria [11]. The application of a combination of psychological pain coping techniques and physical rehabilitation is becoming progressively more demanded of physical therapists, termed psychologically guided physiotherapy [17]. Age, gender, duration of diabetes, blood pressure, and the existence of persistent proteinuria along with retinopathy are risk factors for diabetic nephropathy. It emphasizes how this situation affects the quality of life for those who experience it, mostly because of a variety of physical and health-related problems such as exhaustion, pains, and anaemia [13].

A critical part of complete patient treatment is the physiotherapeutic therapy for paraparesis caused by L2-S1 disc bulging and diabetic neuropathy. This case study emphasises the importance of including physiotherapy, particularly balance training and sensory reeducation, in the management of a complicated disorder with both musculoskeletal and neurovascular components. According to earlier research, balance improves after physical therapy; bilateral lower extremity exercise led to improved gait training [18,19]. Physical therapy objectives vary from patient to patient depending on the individual signs and symptoms [20].

Balance training is critical in the recovery process, particularly when dealing with paraparesis. Impaired balance is a typical issue in people with disc bulges and neuropathy, and it has a substantial impact on their mobility and everyday activities. Balance training physiotherapy techniques aim to improve proprioception, stability, and coordination, reducing the risk of falls and enhancing total functional independence. Sensory reeducation is very important, especially in diabetic neuropathy situations. Gait irregularities and imbalance might be exacerbated by impaired feeling in the lower limbs. Physiotherapists can assist patients in reestablishing a connection with their sensory feedback through tailored sensory reeducation activities, resulting in enhanced motor control.

## Conclusions

A focused treatment plan, highlighting balance training, gait training, and sensory reeducation, along with educating the patient about post-discharge care, proved highly beneficial. Despite initial improvements in the patient's balance and gait, ongoing follow-up care was necessary. This integrated approach's effectiveness emphasizes the potential for positive brain changes and functional gains, providing insights for optimizing rehabilitation outcomes for patients with complicated disorders. The patient acquired awareness of the importance of physiotherapy and the challenges he might face without treatment, even though he couldn't perform his regular activities during the study. 

Continued research and clinical study in this area has the potential to significantly advance our understanding and refine rehabilitation treatments for people suffering from such complex diseases.
